# Comparison of microendoscopic discectomy and percutaneous transforaminal endoscopic discectomy for upper lumbar disc herniation: A protocol for a systematic review and meta-analysis

**DOI:** 10.1097/MD.0000000000027914

**Published:** 2021-11-19

**Authors:** WeiJun Xu, Bingxuan Yang, Xidan Lai, Xinxin Hong, Zihao Chen, Dongqing Yu

**Affiliations:** a Guangdong Chaozhou Health Vocational College, The Second Clinical Medical College of Guangzhou University of Chinese Medicine, Guangdong, China; b Department of Graduate School, Guangzhou University of Chinese Medicine, Guangzhou, China; c Guangdong Provincial Hospital of Chinese Medicine, Guangzhou, China; d The Master Degree Application of Equivalent Educational Level of Guangzhou University of Chinese Medicine, Guangzhou, China; e The Fourth Clinical Medical College of Guangzhou University of Chinese Medicine, Shenzhen, China; f Department of Orthopedics, Traditional Chinese Medical Hospital of Xinjiang Uygur Autonomous Region, Xinjiang, China; g The Third Clinical Medical College of Guangzhou University of Chinese Medicine, Guangzhou, China.

**Keywords:** meta-analysis, microendoscopic discectomy, percutaneous transforaminal endoscopic discectomy, protocol, systematic review, upper lumbar disc herniation

## Abstract

**Background:**

Microendoscopic discectomy (MED) and percutaneous transforaminal endoscopic discectomy (PTED), as two alternative surgical techniques in minimally invasive spine surgery (MISS), are widely conducted in the treatment of upper lumbar disc herniation (ULDH). This study will systematically assess and compare the clinical outcomes of MED and PTED in treating ULDH combining with the meta-analysis.

**Methods:**

All the randomized controlled trials (RCTs) will be searched at the databases including PubMed, EMBASE, Cochrane Library and Web of Science, China National Knowledge Infrastructure (CNKI), Chinese Biomedical Literature Database (CBM), Chinese Scientific Journal Database (VIP), and WANFANG Database from inception to December 2025. The primary outcome will involve Japanese Orthopedic Association (JOA), Oswestry disability index (ODI), and visual analog scale (VAS) scores. The secondary outcomes will be the short-form 36-item (SF-36) health survey questionnaire and the modified MacNab criterion. We will perform data synthesis, subgroup analysis, sensitivity analysis, meta-regression analysis, and the assessment of reporting bias using RevMan 5.3 software.

**Results:**

This systematic review will comprehensively evaluate the clinical outcomes of comparison of MED and PTED in the treatment of ULDH and provide a reliable and high-quality evidence.

**Conclusion:**

The conclusion of this study will elucidate the clinical outcomes of MED compared with PTED and clarify whether PTED generates better clinical effects than MED in treating ULDH.

**PROSPERO registration number:**

CRD 42021244204

## 1 Introduction

Upper lumbar disc herniation (ULDH), with an approximately 5% rare incidence rate, occurs at the upper lumbar spine including L1-L2, L2-L3, and L3-L4 levels.^[1]^ With an ongoing controversy about the definition of what is the specific extent of upper lumbar in lumbar disc herniation, some researchers define the extent of damaged lumbar disc segments as L1–L2 and L2–L3 levels,^[2]^ while others expand it to T12–L1, L1-L2, L2-L3, and L3–4 levels.^[3,4]^ ULDH has unique anatomical structure characteristics including a narrow spinal canal, a location adjacent to the lumbosacral enlargement area of the spinal cord, less distance between the dura and short nerve roots compared with lower lumbar disc herniation (LLDH).^[5]^ In addition, because of the anatomical uniqueness of upper lumbar disc and its surrounding tissue structure, the postoperative efficacy of ULDH was less satisfactory than LLDH.^[1]^ Although there are higher surgical risks and bigger clinical challenges in the treatment of ULDH, it is essential that conducting surgical decompression for ULDH compared with LLDH.^[6]^

Along with the rapid development of surgical techniques, the wide application of minimally invasive spine surgery (MISS) system has been proved to be clinically safe and feasible. Recently, most experienced spine surgeons prefer to perform MISS in order to obtain less trauma, but more satisfactory clinical outcomes in treating lumbar disc herniation.^[7]^ Microendoscopic discectomy (MED) was first reported by Foley et al,^[8]^ the surgical field was viewed using a microendoscope, and the paraspinous muscles were splitted through dilators, which result in less damage to the tissues. MED, confirmed its safety and efficacy by plenty of studies, was well-known as a frequently-used MISS technique over the past few decades.^[9,10]^ Percutaneous endoscopic transforaminal discectomy (PETD) with more minimally invasive and posterior column lumbar structures preserved, was introduced by Yeung et al.^[11]^ Reportedly, PTED without laminectomy and dural traction was an another viable and proper choice for ULDH.^[6,12]^

Since the similar surgical indications of MED and PTED, surgeons face a dilemma in the selection between these two MISS techniques. Numerous relevant studies were conducted to clarify the issue, but their clinical utility were limited by a variety of factors including small sample sizes, variable methodologies, improper confidence intervals, and even conflicting results.^[13–15]^. To our knowledge, there is a lack of systematic reviews and meta-analyses to resolve this debate. Thus, this study is aimed to systematically elucidate the clinical outcomes of MED compared with PTED for ULDH by summarizing the results of published high-quality clinical trials involved both Chinese and English databases, and clarify whether PTED generates better clinical effects than MED in treating ULDH, and provide theoretical guidance and basis in the clinic.

## 2 Methods

### 2.1 Registration

The study will be conducted according to the preferred reporting items for systematic review and meta-analysis protocols (PRISMA-P) statement.^[16]^ This study protocol has been registered in the international prospective register of systematic reviews (PROSPERO) with a trial registration number CRD 42021244204.

### 2.2 Eligibility criteria

#### 2.2.1 Type of study

With the aim of comprehensively elucidating the beneficial effects of different surgical treatments, we will include the randomized controlled trials (RCTs) that investigated the comparison of MED and PTED in the treatment of ULDH. All RCTs will be restricted for the published status and the language of Chinese and English.

#### 2.2.2 Participants

All patients were diagnosed with ULDH by computed tomography (CT) or magnetic resonance imaging (MRI), age 18 to 70 years, regardless of gender, race, region, education, and the course of disease. The patients who suffer from specific diseases including tumor or tuberculosis discitis, ankylosing spondylitis, severe central spinal stenosis, cauda equina syndrome, fracture of lumbar vertebra, and severe osteoporosis will be excluded.

#### 2.2.3 Interventions

The surgical therapy of the control group will be defined as MED, while the experimental group will be set as PTED in eligible studies.

#### 2.2.4 Outcomes

The primary outcome will involve Japanese Orthopedic Association (JOA), Oswestry disability index (ODI), and visual analog scale (VAS) scores in this study.^[17,18]^ The secondary outcomes will be short-form 36-item (SF-36) health survey questionnaire and the modified MacNab criterion.^[12,17]^

### 2.3 Ineligibility criteria

We will exclude non-RCTs such as case reports, observational researches, conference articles, and reviews.

### 2.4 Search strategy

The studies will be searched from inception to December 2025 in both English and Chinese databases including PubMed, EMBASE, Cochrane Library and Web of Science, China National Knowledge Infrastructure (CNKI), Chinese Biomedical Literature Database (CBM), Chinese Scientific Journal Database (VIP), and WANFANG Database. The search topics will consist of P+I+C+O+S in order to improve the rate of literature retrieval. The key terms in the literature retrieval are shown in Table 1. Meanwhile, the fields such as MeSH terms, keywords, abstract and title will be used to search relevant studies in the various databases.

**Table 1:** Search items.

PICOS	Key search terms
Participants	Upper Lumbar Disc Herniation; Upper Lumbar Disk Herniation; Herniated Disc; Disc, Herniated; Disc Displacement, Intervertebral; Intervertebral Disc Displacements; Prolapsed Disc; Disc, Prolapsed; Slipped Disc; Disc, Slipped.
Interventions	Percutaneous Transforaminal Endoscopic Discectomy; Percutaneous Endoscopic Discectomy; Transforaminal Endoscopic Discectomy; Percutaneous Discectomy; Percutaneous Nucleotomy.
Comparisons	Microendoscopic Discectomy; Endoscopic Discectomy; Surgery, Minimally Invasive; Surgical Procedure, Minimal; Surgical Procedure, Minimal Access; Surgical Procedure, Minimally Invasive; Procedure, Minimally Invasive Surgical; Procedure, Minimal Surgical; Procedure, Minimal Access Surgical; Minimal Surgical Procedure; Minimal Access Surgical Procedures; Minimally Invasive Surgery; Minimally Invasive Surgical Procedure.
Outcomes	JOA Scores; ODI Scores; VAS Scores; 36-SF; MacNab criterion.
Study design	Randomized Controlled Trial; Controlled Clinical Trial; Randomized; Random Sampling.

### 2.5 Study selection and data extraction

#### 2.5.1 Study selection

**Figure 1:**
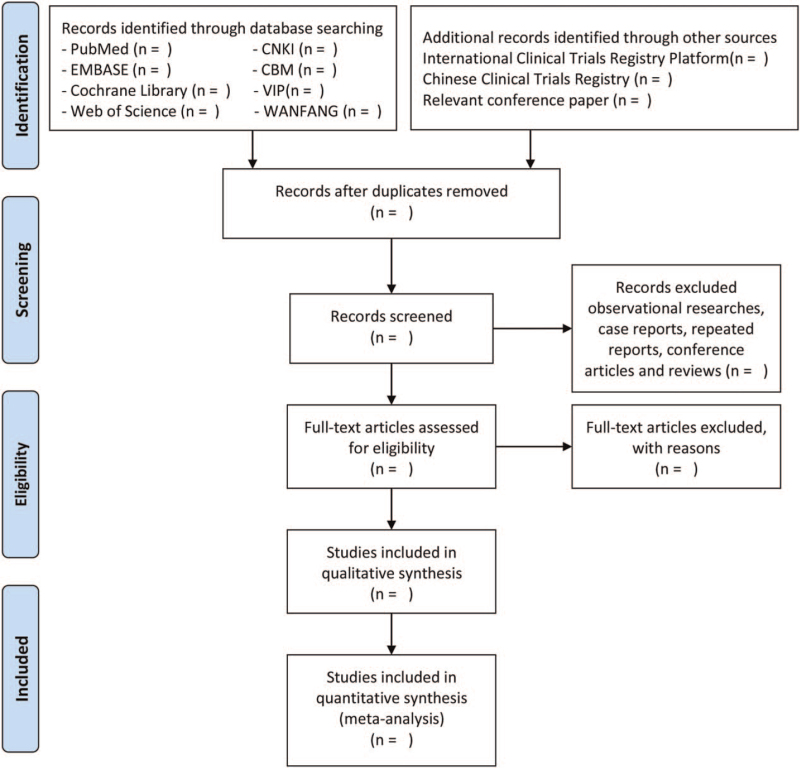
PRISMA flow diagram of studies identified process in the systematic review.

Two reviewers will take charge of the initial screening process independently, then remove repeated data and evident unqualified literatures. The third reviewer will assess the full text of divergent literatures comprehensively and organize discussion group to deal with disagreement. The excluded reasons for ineligible literatures will be recorded in the second filter stage. The selection process of eligible studies is illustrated by a PRISMA flow diagram in Fig. 1.

#### 2.5.2 Data extraction

All the data including title, first author, publication date, country or region, study design, sample size, age, gender, disease course, intervention, outcomes, follow-up, complications, and recurrences will be independently extracted from eligible literatures by two researchers. The third researcher, as an arbitrator, will review those data once again and then work with the expert group to resolve differences.

#### 2.5.3 Processing missing data

If the data of the study is incomplete, we will contact the lead author of the study via email or phone to obtain the missing data. If the data is unavailable, the study will be excluded and the impact of missing data will be indicated in the discussion.

### 2.6 Risk of bias assessment

Our reviewers will evaluate the risk of bias among the included studies using the Cochrane Collaboration recommendations.^[19]^ Seven detailed items of assessment are as followed: stochastic sequence generation, allocation concealment, blinding of participants and personnel, blinding of outcome assessments, fragmentary outcome data, selective reporting and other sources of bias. The bias of above-mentioned items can be divided into low, unclear, and high risk. If the disagreements emerge, we will discuss with a third reviewer to resolve differences.

### 2.7 Statistical analysis

Data synthesis and statistical analysis will be performed using RevMan 5.3 software provided by the Cochrane Collaboration. We will analyze the data using odd ratio or risk ratio with 95% confidence interval (CI) for dichotomous outcomes, while we will compute weighted mean difference (WMD) or standardized mean difference (SMD) with 95% CI for continuous outcomes. The *I*-squared statistic will be calculated to assess the heterogeneity among all the researches, the selection of analytical model will also depend on the value of *I*^2^.^[20]^ If *I*^2^ <50%, suggesting there is nonsignificant heterogeneity, we are supposed to choose a fixed-effect model. On the contrary, if *I*^2^ ≥50%, demonstrating that heterogeneity is substantial significant, we will conduct a meta-analysis using a random-effect model.

### 2.8 Subgroup analysis

If there is substantial heterogeneity in the included papers, we will perform subgroup analysis including: age, gender, race, onset time, duration of disease, phenotype of ULDH, detailed procedure of the operation and other related factors.

### 2.9 Sensitivity analysis

In order to identify the quality of researches and determine the reliability of conclusions, we will conduct sensitivity analysis based on sample size, statistical model, heterogeneity quality, the impact of missing data, and methodological quality.^[21]^

### 2.10 Assessment of reporting biases

If the quantity of pooled studies is adequate in the meta-analysis (n ≥ 10), we will determine symmetry using Begg or Egger test on a funnel plot which is made to evaluate reporting bias.

### 2.11 Test sequential experiment

Sample size analysis will be performed through trial sequential analysis (TSA) to verify the reliability of analysis results and eliminate the false positive probabilities.^[22]^

### 2.12 Quality of evidence

We will assessed the quality of evidence by the Grading of Recommendations Assessment, Development, and Evaluation (GRADE) approach, which grades the scientific evidence into four levels: very low, low, moderate, and high.^[23]^ The limitations of study design, inaccuracies, inconsistencies, and reporting biases will also be investigated by GRADE approach.

### 2.13 Ethics and dissemination

All the data will be derived from the published studies in the different databases, which are not directly from the patient personal data. Therefore, the ethical approval is not required. This protocol will offer a credible theoretical foundation for exploring which surgical technique is better between MED and PTED in treatment of ULDH. The findings of systematic review will be disseminated in a peer-reviewed journal, which can provide surgeon a better surgical strategy and offer basis for the clinical treatment of ULDH.

## 3 Discussion

It is well-known that the anatomical structure of the upper lumbar spine is characterized by larger dural sac and narrower spinal canal compared with lower lumbar spine, and some other structures including lumbar nerve roots and cauda equinus can also lead to compression and disorder of upper lumbar disc.^[24]^ Moreover, the nerve roots without any innervated specific muscles result in nonspecific clinical symptoms and neurological signs, which can cause the misdiagnosis of ULDH.^[25]^ The clinic outcome of ULDH is less satisfactory compared with LLDH, on account of anatomical complexity and high misdiagnosis rate. With the progress and development of MISS system, increasing surgical techniques and operation method with a variety of surgical approaches can be selected for ULDH to obtain more desirable clinical outcomes.^[6,26]^ Currently, MED and PTED are two different surgical techniques for the treatment of lumbar disc herniation, presenting great clinic outcomes in the improvement of signs, symptoms, VAS scores, ODI scores, SF-36 scores.^[13]^ However, there is no systematic review or meta-analysis about the comparison of PTED and MED for ULDH that has been conducted systematically yet. Therefore, we determine to evaluate the clinic outcomes of MED compared with PTED for the treatment of ULDH and perform a systematic review and meta-analysis based on the high-quality RCTs to offer clinic basis for surgical treatments and verify whether PTED provides better clinical outcomes than MED. Along with the widespread application of surgical techniques and the rapid development of researches, some new studies will not be included, and it is hardly immune to limitations such as heterogeneity and publishing bias, so it is crucial to analyze more results and researches.

## Author contributions

**Conceptualization:** WeiJun Xu, Bingxuan Yang, Dongqing Yu.

**Data curation:** WeiJun Xu, Bingxuan Yang, Xidan Lai, Xinxin Hong.

**Formal analysis:** WeiJun Xu, Bingxuan Yang, Zihao Chen.

**Investigation:** Bingxuan Yang, Xidan Lai, Xinxin Hong.

**Methodology:** WeiJun Xu, Bingxuan Yang, Xidan Lai, Dongqing Yu.

**Software:** WeiJun Xu, Bingxuan Yang, Zihao Chen.

**Supervision:** Dongqing Yu.

**Validation:** Dongqing Yu.

**Writing – original draft:** WeiJun Xu, Bingxuan Yang.

**Writing – review & editing:** WeiJun Xu, Bingxuan Yang, Dongqing Yu.

## References

[1] Sanderson SP Houten J Errico T Forshaw D Bauman J Cooper PR. The unique characteristics of “upper” lumbar disc herniations. Neurosurgery 2004;55:385–9. 15271245 10.1227/01.neu.0000129548.14898.9b

[2] Kim D-S Lee J-K Jang J-W Ko B-S Lee J-H Kim S-H. Clinical features and treatments of upper lumbar disc herniations. J Korean Neurosurg S 2010;48:119. 10.3340/jkns.2010.48.2.119 PMC2941853 20856659

[3] Yüce I Kahyaoğlu O Mertan P Çavuşoğlu H Aydin Y. Analysis of clinical characteristics and surgical results of upper lumbar disc herniations. Neurochirurgie 2019;65:158–63. 31100349 10.1016/j.neuchi.2019.04.002

[4] Hsu K Zucherman J Shea W. High lumbar disc degeneration. Incidence and etiology. Spine (Phila Pa 1976) 1990;15:679–82. 2218715 10.1097/00007632-199007000-00012

[5] Lee D-S Park K-S Park M-S. The comparative analysis of clinical characteristics and surgical results between the upper and lower lumbar disc herniations. J Korean Neurosurg S 2013;54:379. 10.3340/jkns.2013.54.5.379 PMC3873349 24379943

[6] Ahn Y Lee S-H Lee JH Kim JU Liu WC. Transforaminal percutaneous endoscopic lumbar discectomy for upper lumbar disc herniation: clinical outcome, prognostic factors, and technical consideration. Acta Neurochir 2009;151:199–206. 19229467 10.1007/s00701-009-0204-x

[7] Sairyo K Chikawa T Nagamachi A. State-of-the-art transforaminal percutaneous endoscopic lumbar surgery under local anesthesia: discectomy, foraminoplasty, and ventral facetectomy. J Orthop Sci 2018;23:229–36. 29248305 10.1016/j.jos.2017.10.015

[8] Foley KT Smith MM Rampersaud YR. Microendoscopic approach to far-lateral lumbar disc herniation. Neurosurg Focus 1999;7:E7. 10.3171/foc.1999.7.6.6 16918212

[9] Hubbe U Franco-Jimenez P Klingler J-H Vasilikos I Scholz C Kogias E. Minimally invasive tubular microdiscectomy for recurrent lumbar disc herniation. J Neurosurg-Spine 2016;24:48–53. 26384131 10.3171/2015.4.SPINE14883

[10] Tsutsumimoto T Yui M Uehara M Ohta H Kosaku H Misawa H. A prospective study of the incidence and outcomes of incidental dural tears in microendoscopic lumbar decompressive surgery. Bone Joint J 2014;96:641–5. 24788499 10.1302/0301-620X.96B5.32957

[11] Yeung AT Tsou PM. Posterolateral endoscopic excision for lumbar disc herniation: surgical technique, outcome, and complications in 307 consecutive cases. Spine (Phila Pa 1976) 2002;27:722–31. 11923665 10.1097/00007632-200204010-00009

[12] Li Z Zhang C Chen W. Percutaneous endoscopic transforaminal discectomy versus conventional open lumbar discectomy for upper lumbar disc herniation: a comparative cohort study. Biomed Res Int 2020;2020:01–7. 10.1155/2020/1852070 PMC7072112 32190653

[13] Chen Z Zhang L Dong J. Percutaneous transforaminal endoscopic discectomy versus microendoscopic discectomy for lumbar disc herniation: two-year results of a randomized controlled trial. Spine (Phila Pa 1976) 2020;45:493–503. 31703056 10.1097/BRS.0000000000003314

[14] Liu X Yuan S Tian Y. Comparison of percutaneous endoscopic transforaminal discectomy, microendoscopic discectomy, and microdiscectomy for symptomatic lumbar disc herniation: minimum 2-year follow-up results. J Neurosurg-Spine 2018;28:317–25. 29303471 10.3171/2017.6.SPINE172

[15] Abudurexiti T Qi L Muheremu A Amudong A. Micro-endoscopic discectomy versus percutaneous endoscopic surgery for lumbar disk herniation. J Int Med Res 2018;46:3910–7. 29900752 10.1177/0300060518781694 PMC6136013

[16] Moher D Shamseer L Clarke M. Preferred reporting items for systematic review and meta-analysis protocols (PRISMA-P) 2015 statement. Syst Rev-London 2015;4:01–9. 10.1186/2046-4053-4-1 PMC4320440 25554246

[17] Fujiwara A Kobayashi N Saiki K Kitagawa T Tamai K Saotome K. Association of the Japanese Orthopaedic Association score with the Oswestry disability index, Roland-Morris disability questionnaire, and short-form 36. Spine (Phila Pa 1976) 2003;28:1601–7. 12865852

[18] Chiarotto A Maxwell L Ostelo R Boers M Tugwell P Terwee C. Measurement properties of visual analogue scale, numeric rating scale, and pain severity subscale of the brief pain inventory in patients with low back pain: a systematic review. J Pain 2019;20:245–63. 30099210 10.1016/j.jpain.2018.07.009

[19] Higgins JP Altman DG Gøtzsche PC. The Cochrane Collaboration's tool for assessing risk of bias in randomised trials. BMJ-Brit Med J 2011;343. 10.1136/bmj.d5928 PMC3196245 22008217

[20] Higgins JP Thompson SG Deeks JJ Altman DG. Measuring inconsistency in meta-analyses. BMJ-Brit Med J 2003;327:557–60. 10.1136/bmj.327.7414.557 PMC192859 12958120

[21] Egger M Smith GD Schneider M Minder C. Bias in meta-analysis detected by a simple, graphical test. BMJ-Brit Med J 1997;315:629–34. 10.1136/bmj.315.7109.629 PMC2127453 9310563

[22] Wetterslev J Thorlund K Brok J Gluud C. Trial sequential analysis may establish when firm evidence is reached in cumulative meta-analysis. J Clin Epidemiol 2008;61:64–75. 18083463 10.1016/j.jclinepi.2007.03.013

[23] Puhan MA Schünemann HJ Murad MH. A GRADE working group approach for rating the quality of treatment effect estimates from network meta-analysis. BMJ-Brit Med J 2014;349. 10.1136/bmj.g5630 25252733

[24] Wiltse LL Berger PE McCulloch JA. A system for reporting the size and location of lesions in the spine. Spine (Phila Pa 1976) 1997;22:1534–7. 9231975 10.1097/00007632-199707010-00023

[25] Kido T Okuyama K Chiba M. Clinical diagnosis of upper lumbar disc herniation: pain and/or numbness distribution are more useful for appropriate level diagnosis. J Orthop Sci 2016;21:419–24. 27053156 10.1016/j.jos.2016.03.003

[26] Son S Lee SG Kim WK Ahn Y. Advantages of a microsurgical translaminar approach (keyhole laminotomy) for upper lumbar disc herniation. World Neurosurg 2018;119:e16–22. 29902597 10.1016/j.wneu.2018.06.004

